# Surgical management of atypically located thoracic schwannomas: a single-center case series

**DOI:** 10.1007/s13304-026-02528-7

**Published:** 2026-02-06

**Authors:** Süleyman Gökalp Güneş, Yusuf Kahya, Şeyma Nur Turan, Saliha Yılmaz, Ahmet Altıntaş, Bülent Mustafa Yenigün

**Affiliations:** Department of Thoracic Surgery, Ankara University Faculty of Medicine, İbni Sina Hospital, Talatpaşa Blv, Altındag, Ankara, Turkey

**Keywords:** Thoracic schwannoma, Atypical location, Mediastinum, RATS, VATS, Case series

## Abstract

Schwannomas are benign nerve sheath tumors most commonly located in the posterior mediastinum. However, atypically located thoracic schwannomas are rare and may pose challenges in diagnosis and surgical management. In this retrospective case series, 14 patients who underwent surgical treatment for atypically located thoracic schwannomas between 2011 and 2025 were evaluated. Demographic, clinical, radiological, surgical, and histopathological data were analyzed. The associations between tumor location and clinical or surgical variables were assessed using Fisher’s exact test and the Chi-square test. The mean age of the patients was 51.4 years (range: 24–77), with a predominance of male cases. Tumor locations were classified as supraclavicular (*n* = 4), anterior/middle mediastinum (*n* = 4), infraclavicular (*n* = 2), chest wall (*n* = 3), and lung parenchyma (*n* = 1). Pain was the most common presenting symptom. All patients underwent curative (R0) surgical resection. Surgical approaches included VATS (*n* = 4), RATS (*n* = 1), thoracotomy (*n* = 3), and modified open techniques (*n* = 6). No statistically significant association was found between tumor location and complication rates, surgical method, or length of hospital stay. No recurrences were observed during a mean follow-up period of 48 months. Although rare, atypically located thoracic schwannomas should be considered in the differential diagnosis of intrathoracic masses. With accurate preoperative imaging and proper surgical planning, these lesions can be safely and completely resected, resulting in favorable clinical outcomes.

**Graphical abstract:**

**Figure:**
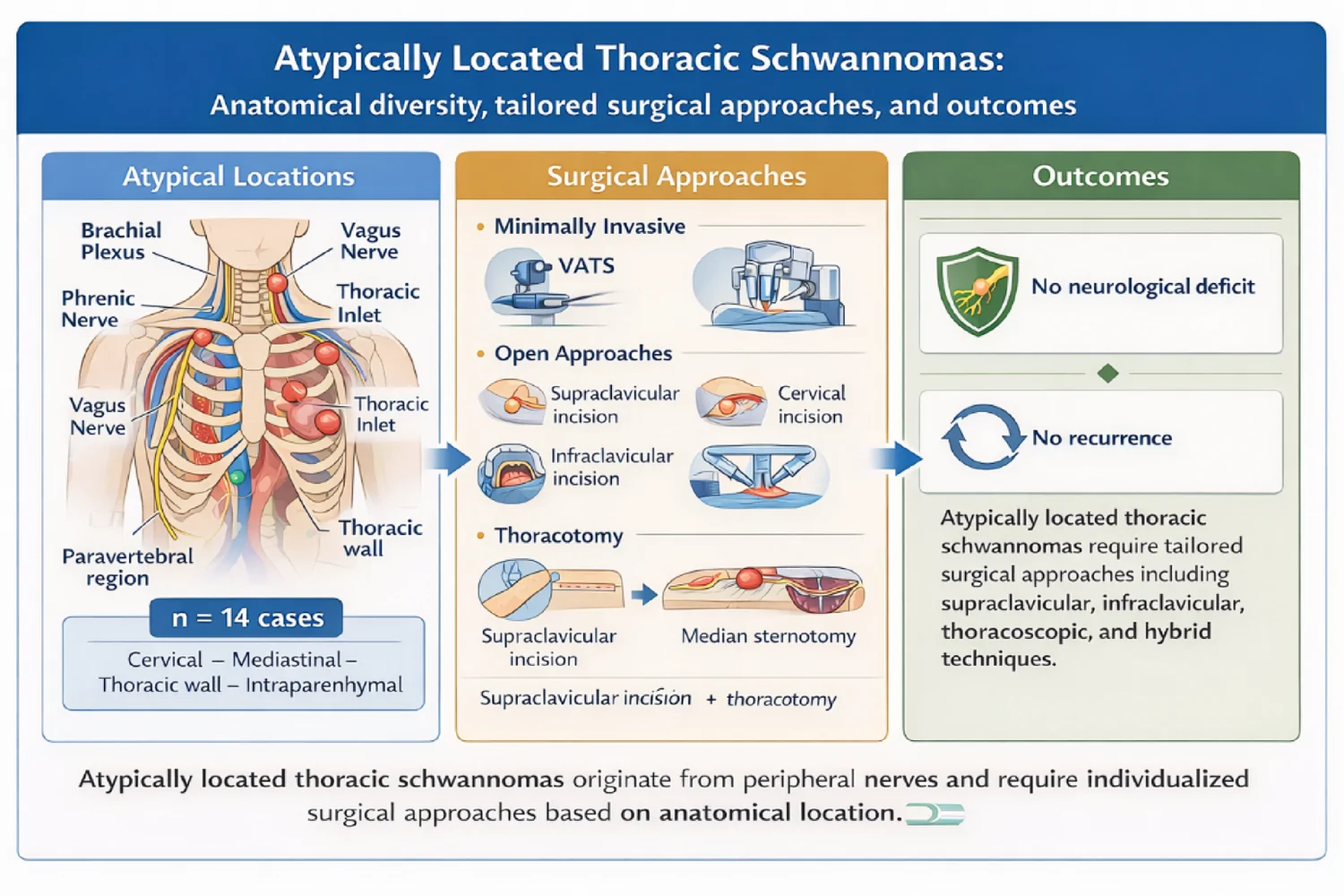

## Introduction

Schwannomas are the most common benign peripheral neurogenic tumors, originating from Schwann cells within the nerve sheaths, and account for approximately 1–2% of all thoracic tumors. Peripheral nerve sheath tumors are classified by the WHO based on histopathological features, and malignant transformation is rare [1, 2]. Schwannomas are more commonly seen in adults. Diagnosis is usually based on their thoracic location using advanced radiologic imaging. Preoperative biopsy is required only in selected cases. Contrast-enhanced CT is the standard imaging method. MRI is useful for assessing possible invasion of adjacent structures. 18F-FDG positron emission tomography (PET-CT) may also assist in the differential diagnosis. For tumors near the spinal canal—especially at T8–L1—preoperative identification of the Adamkiewicz artery is important. Contrast-enhanced studies such as CT angiography help reduce the risk of vascular complications [3].

Schwannomas commonly arise in the flexor surfaces of the extremities, the neck, mediastinum, retroperitoneum, and posterior spinal roots. Within the thoracic cavity, they most frequently originate from the posterior mediastinum, although they may occasionally be found in atypical locations. Schwannomas account for approximately 12–21% of all mediastinal masses and typically arise from spinal nerve roots or the sympathetic chain in the posterior mediastinum. Less commonly, they may originate from the vagus or recurrent laryngeal nerves in the middle mediastinum, or from the phrenic nerve—and rarely the vagus nerve—in the anterior mediastinum [4]. While thoracic schwannomas are most often located in the posterior mediastinum, occurrences in the anterior or middle mediastinum are rare. Due to their rarity, schwannomas in these atypical thoracic locations have been described in only a limited number of case reports in the literature. In this study, we aimed to present a series of schwannoma cases located in uncommon thoracic sites.

## Materials and methods

A total of 84 patients who underwent surgery in our clinic between 2011 and 2025 for thoracic schwannomas located in atypical sites were retrospectively reviewed. Seventy of these patients had tumors located in the posterior mediastinum or paravertebral region, which are the most commonly reported locations for schwannomas in the literature. Atypical location was defined as any thoracic schwannoma arising outside the posterior mediastinum and paravertebral region, which represent the typical sites of origin. Accordingly, tumors located in the supraclavicular region, anterior or middle mediastinum, infraclavicular area, chest wall (intercostal spaces), and pulmonary parenchyma were included. Patients with tumors originating from the posterior mediastinum, paravertebral sulcus, or spinal nerve roots—considered typical locations were excluded from the study.

Clinical presentations, demographic characteristics, radiological findings, surgical approaches, and recurrence status were recorded for each patient. All patients underwent routine thorax computed tomography (CT) scanning. In cases where invasion of adjacent structures was suspected, further evaluation was performed using magnetic resonance imaging (MRI). Patients with differential diagnostic considerations involving other tumor types were additionally assessed with fluorodeoxyglucose positron emission tomography (FDG-PET/CT). For a small subset of patients in whom imaging studies could not yield a definitive diagnosis, preoperative histopathological confirmation was obtained via transthoracic fine-needle aspiration biopsy.

The mean follow-up duration was 48 months, ranging from 6 to 120 months. It was observed that patients with longer follow-up durations were being monitored for reasons unrelated to schwannoma, and no recurrence was detected in any of the followed patients.

### Statistical analysis

Statistical analyses were performed using IBM SPSS Statistics for Windows, Version 25.0 (IBM Corp., Armonk, NY, USA). Fisher’s exact test and the Chi-square test were used to evaluate associations between tumor localization and categorical variables such as symptom presence, complication occurrence, tumor size, surgical approach, and length of hospital stay. Fisher’s exact test was applied to 2 × 2 contingency tables, while the Chi-square test was used for larger contingency tables. A *p*-value of < 0.05 was considered statistically significant.

## Findings and results

The mean age of patients with atypically located thoracic schwannomas was 51.4 years (range: 24–77), with 42.8% (*n* = 6) being female and 57.2% (*n* = 8) male. Pain, depending on tumor localization, was the most common presenting symptom, followed by dyspnea and cough. In three patients, incidental masses were detected during radiological imaging performed for oncologic follow-up. One patient was investigated due to recurrent shoulder dislocation, leading to the diagnosis of a thoracic mass.

Tumors were located in the supraclavicular region (*n* = 4), anterior/middle mediastinum (*n* = 4), infraclavicular area (*n* = 2), chest wall (*n* = 3), and lung parenchyma (*n* = 1). All patients underwent curative R0 resections (Table 1). Tumor sizes ranged from 20 to 135 mm. Postoperative pneumonia was observed in two patients. The mean chest tube drainage was 80 mL (range: 20–150 mL), and the average length of hospital stay was 3 days (range: 2–5 days). No major complications aside from two cases of mild pneumonia, and no reoperations or adjuvant therapies were required.

**Table 1:** Patients characteristics

Patient no	Gender (F/M)	Age	Symptoms	Tumor location	Tumor size (mm)	Procedure
1	F	24	Pain and numbness in the left upper extremity	Left Supraclavicular, C7-T1	65 × 50 mm	Modified cervical-supraclavicular J insicion
2	F	25	Exertional dyspnea	Anterior and middle mediastinum, left hemithorax	135 × 100 mm	Median sternotomy
3	M	31	Recurrent shoulder dislocation	Right infraklavicular, axillary	75 × 50 mm	Modified infraclavicular insicion
4	M	47	Covid symptoms	Right supraclavicular, paravertabral	35 × 25 mm	Modified supraclavicular insicion
5	M	46	Cough	Precarinal, paratraceal	35 × 25 mm	Right RATS
6	F	55	Left flank pain	Thorax wall	40 × 40 mm	Left VATS
7	M	65	Cough	Right superior mediastinum, C7–T2	120 × 80 mm	Modified right supraclavicular insicion + Right thoracotomy
8	M	52	Neck pain	Left infraclavicular	90 × 60 mm	Supraclavicular J insicion
9	F	77	Oncologycal follow-up due to papillary thyroid carcinoma	Precarinal, middle mediastinum	50 × 35 mm	Right VATS
10	M	58	Oncologycal follow-up due to malign schwannoma (humerus)	Left lower lobe parencyma	20 × 10 mm–15 × 10 mm	Left thoracotomy
11	M	47	Chest pain	Thorax Wall	35 × 20 mm	Left VATS
12	M	52	Chest pain	Thorax Wall	25 × 15 mm	Right VATS
13	F	62	Neck and upper extremity pain	Left supraclavicular- pectoral	85 × 75 mm	Modified pectoral insicion
14	F	79	Oncologycal follow up due to lymphoma	Right supraclavicular	30 × 25 mm	Modified supraclavicular insicion

In our series, no postoperative neurological deficits were detected, even in tumors located near the brachial plexus, vagus nerve, or phrenic nerve. We attribute this favorable outcome to the routine use of intraoperative nerve stimulation and meticulous dissection performed with careful attention to regional anatomy.

No statistically significant associations were identified between tumor localization and any of the evaluated clinical or surgical variables, including symptom presence, postoperative complications, tumor size (> 5 cm), surgical approach, or length of hospital stay (all *p* > 0.05). Similarly, no significant relationships were found between PET positivity and tumor size or between tumor size and complication occurrence. Although certain clinical patterns were noted, none reached statistical significance, likely due to the limited sample size (Table 2).

**Table 2:** Summary of associations between tumor localization and clinical/surgical variables

Variable	*p* Value
Symptom presence	0.35
Postoperative complications	0.13
Tumor size (> 5 cm)	0.14
Surgical approach (VATS/RATS/Thoracotomy)	0.21
Hospital stay (> 3 days)	0.32
FDG-PET positivity vs tumor size	1.00
Complications vs tumor size	0.46

Associations between categorical variables were assessed using Fisher’s exact test or the Chi-square test, selected based on expected cell counts

## Case series

A 24-year-old female presented with pain and numbness in the left upper arm. Thoracic CT and MRI revealed a 65 × 50 mm supraclavicular mass at the C7–T1 level, extending into the left neural foramen and compressing the subclavian artery. Histopathological analysis of ultrasound-guided biopsy was consistent with schwannoma. The mass was completely excised through a modified cervical-supraclavicular J incision. No recurrence was observed at the 6-month follow-up.

A 25-year-old female with exertional dyspnea underwent thoracic CT and MRI, which demonstrated a heterogeneous 135 × 100 mm mass extending from the left thyroid lobe to the level of the pulmonary artery. The mass was in contact with the subclavian artery, main pulmonary artery, and left pulmonary artery, and showed areas of calcification and fat density. The lesion was resected via median sternotomy in collaboration with cardiovascular surgery. The patient was discharged on postoperative day 5, and no recurrence was observed at the 8-month follow-up.

In a patient presenting with the atypical symptom of recurrent shoulder dislocation, thoracic imaging revealed a 75 × 50 mm mass located between the humeral head and the thoracic wall in the right infraclavicular region. The mass was resected via a modified infraclavicular incision. No recurrence was noted at the 12-month follow-up.

One patient being evaluated for COVID-19 pneumonia was found to have an incidental paravertebral paraesophageal mass at the C7–T1 level, which was excised through a modified supraclavicular incision. In three patients presenting with chest pain, chest wall tumors located at the intercostal spaces were resected via video-assisted thoracoscopic surgery (VATS). One patient with a parasternal supraclavicular lesion underwent resection through a supraclavicular incision. Another patient with a large supraclavicular tumor (85 × 75 mm) underwent resection via a modified pectoral incision.

A 58-year-old male previously treated for malignant schwannoma of the humerus underwent thoracic CT for oncologic follow-up, which revealed two parenchymal nodules in the left lower lobe measuring 20 mm and 15 mm, with SUVmax values of 12.0 and 4.9 on PET-CT, respectively. The patient underwent left thoracotomy and multiple wedge resections. Histopathological evaluation confirmed metastatic malignant peripheral nerve sheath tumor. No thoracic recurrence was observed during the 48-month follow-up.

**Fig. 1:**
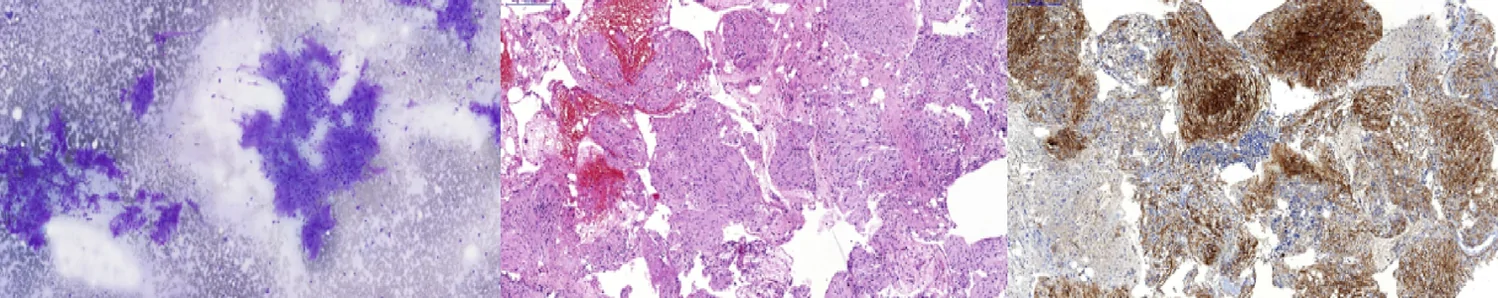
Preoperative endobronchial ultrasound (EBUS) image and digital histopathological section obtained from the resected tumor specimen. **a** Smear from endobronchial ultrasound-guided fine-needle aspiration (EBUS-FNA) showing scattered spindle-shaped mesenchymal cells within a collagenized matrix (May-Grünwald-Giemsa stain, × 11.6 magnification). **b** Cell block preparation from cytologic material revealing a spindle cell mesenchymal tumor (Hematoxylin and Eosin stain, × 11.6 magnification). **c** Immunohistochemical staining of the cell block showing diffuse S-100 protein positivity in tumor cells

A 77-year-old female with a history of papillary thyroid carcinoma had a 50 × 35 mm pre-carinal mass in the middle mediastinum with a SUVmax of 9.4 on PET-CT. Endobronchial ultrasound (EBUS)-guided transbronchial needle biopsy revealed features consistent with schwannoma. The lesion was resected via right thoracotomy. The patient was followed for 10 years postoperatively with no evidence of recurrence (Fig. 1).

One patient with a 35 × 25 mm mass located in the pre-carinal region underwent resection via robot-assisted thoracic surgery (RATS).

A 52-year-old male presented with neck and left arm pain accompanied by symptoms consistent with thoracic outlet syndrome. Thoracic CT and CT angiography revealed a 90 × 60 mm supraclavicular-retropectoral mass that caused approximately 50% narrowing of the left subclavian artery during Adson’s maneuver. MRI showed that the mass was in contact with the brachial plexus. The mass was excised via a supraclavicular J incision. No recurrence was observed during the 48-month follow-up.

**Fig. 2:**
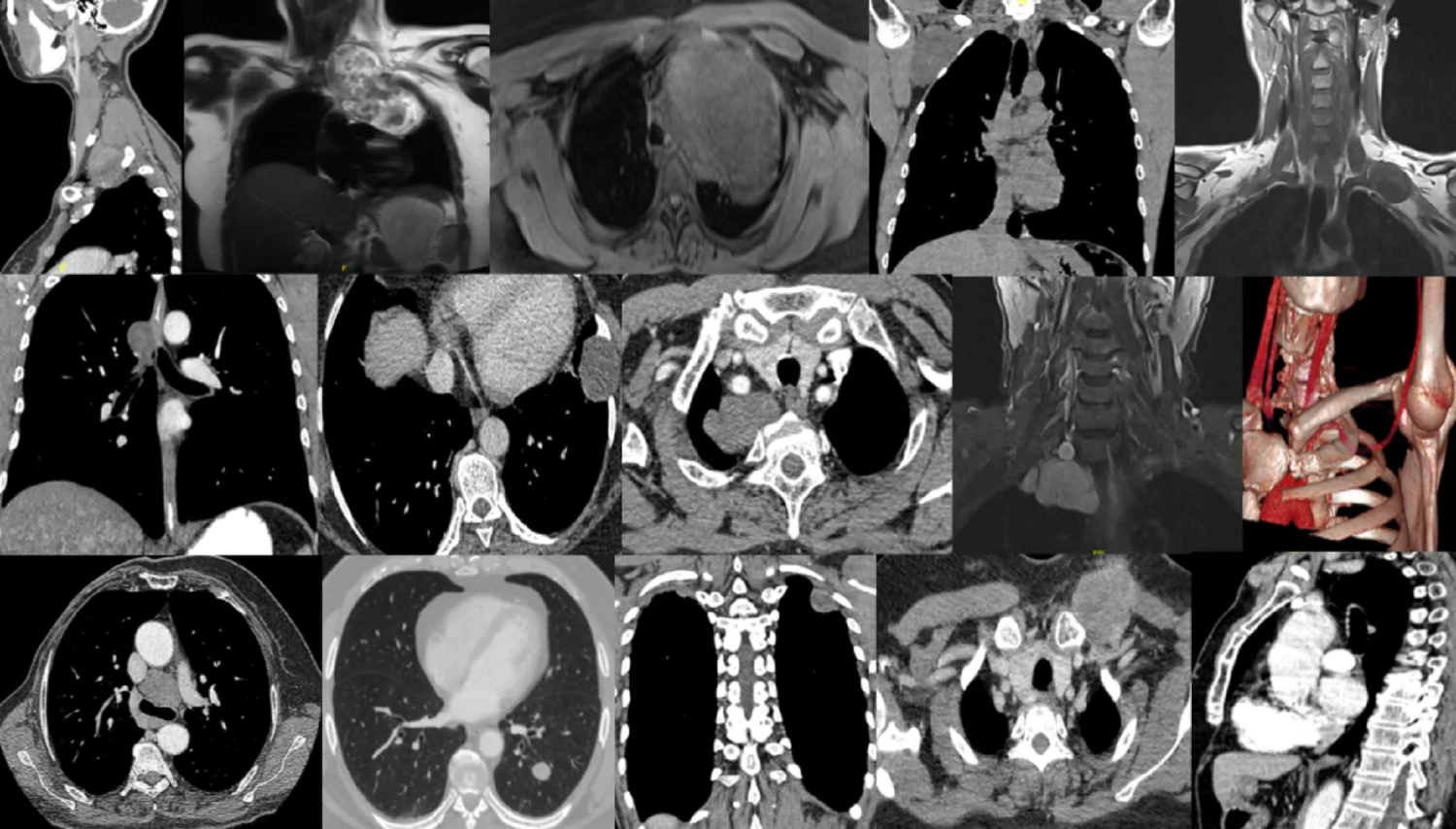
Preoperative thoracic computed tomography (CT) and magnetic resonance imaging (MRI) scans of selected cases

**Fig. 3:**
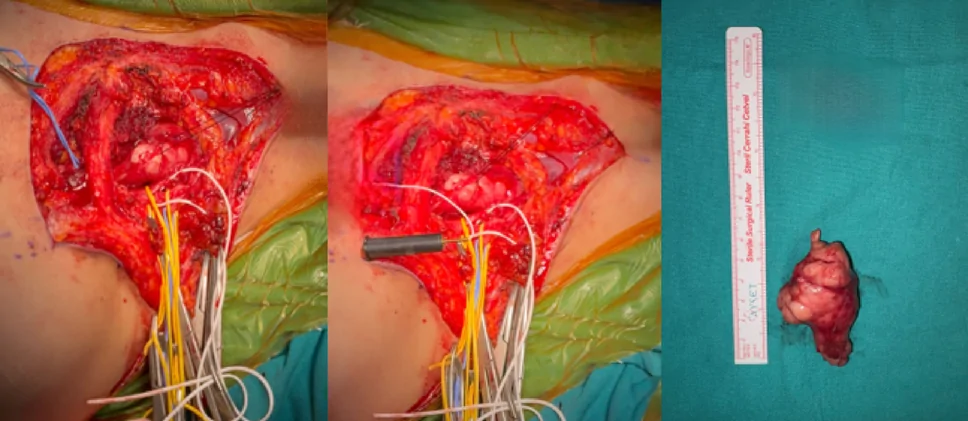
Intraoperative view showing the surgical incision, anatomical structures, and the excised tumor specimen

A 65-year-old male presenting with persistent cough was found on PET-CT to have a hypermetabolic mass (SUVmax: 3.5) extending from the superior mediastinum to the cervical level (C7), located posterior to the subclavian vein and right vertebral artery. The lesion measured 120 × 80 mm and demonstrated close proximity to the C7–T1 and T1–T2 neural foramina. It appeared isointense on T1-weighted MRI and hyperintense on T2-weighted imaging, with restricted diffusion. The tumor was completely excised through a combined modified supraclavicular incision and right thoracotomy. The patient was followed for 5 postoperative days and showed no evidence of recurrence at the 48-month follow-up (Figs. 2, 3).

## Discussion

Surgical resection remains the gold standard treatment for schwannomas, provided that appropriate preoperative evaluations are conducted [5]. Although clinical presentation varies depending on tumor location and size, patients frequently experience chest or back pain. However, as demonstrated in our case series, atypical tumor locations may lead to unusual symptoms such as neck or arm pain, or even recurrent shoulder dislocation [6]. For instance, in a case report by Jang et al., a posterior thoracic wall schwannoma presented with dyspnea secondary to total pleural effusion [7]. These tumors are typically seen in young to middle-aged adults; however, an increasing number of cases are being diagnosed in adolescent patients, likely due to advances in diagnostic imaging and more frequent clinical evaluation [8].

**Fig. 4:**
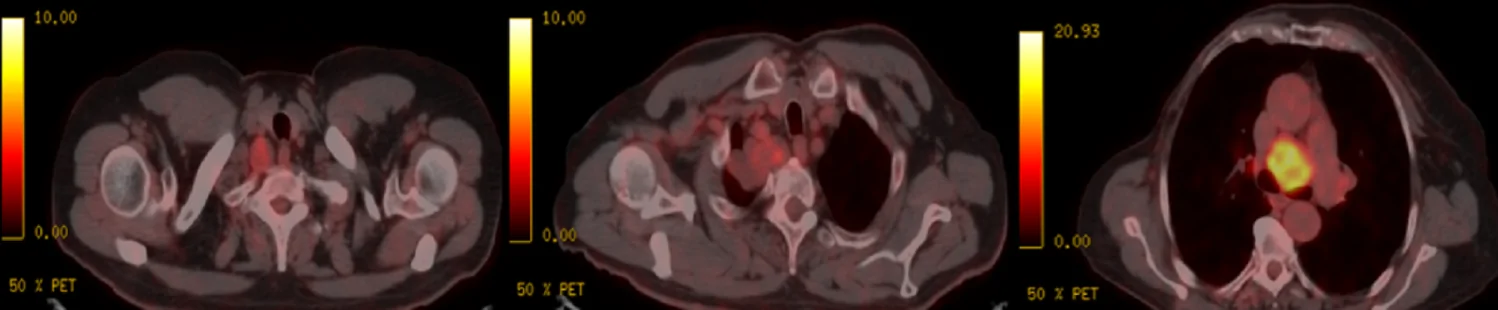
Preoperative 18F-FDG PET-CT images obtained for differential diagnosis in selected cases

CT is routinely used for diagnosis. MRI provides additional detail when invasion of adjacent structures is suspected. Moreover, fluorodeoxyglucose positron emission tomography (FDG-PET/CT) can be a valuable adjunct in the differential diagnosis and plays an important role in preoperative surgical planning for achieving complete resection [9] (Fig. 4).

**Fig. 5:**
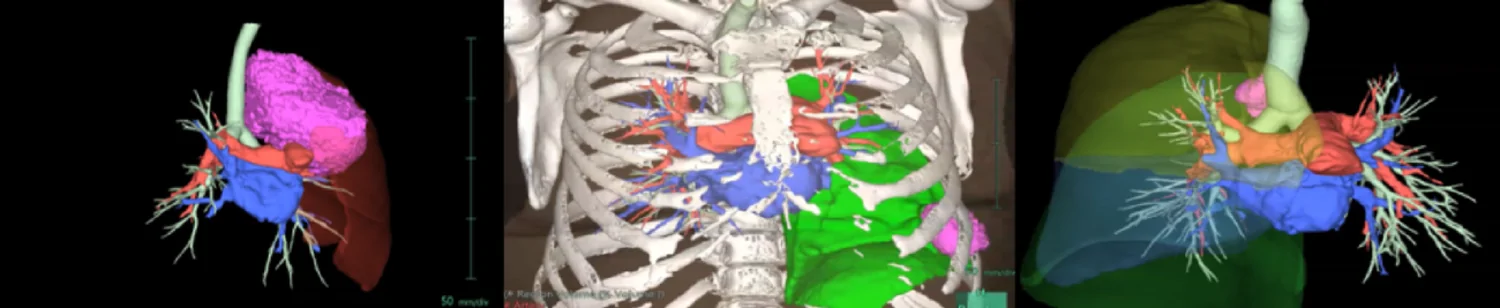
Synapse-based 3D modeling images from selected cases illustrating anatomical relationships of mediastinal masses

**Fig. 6:**
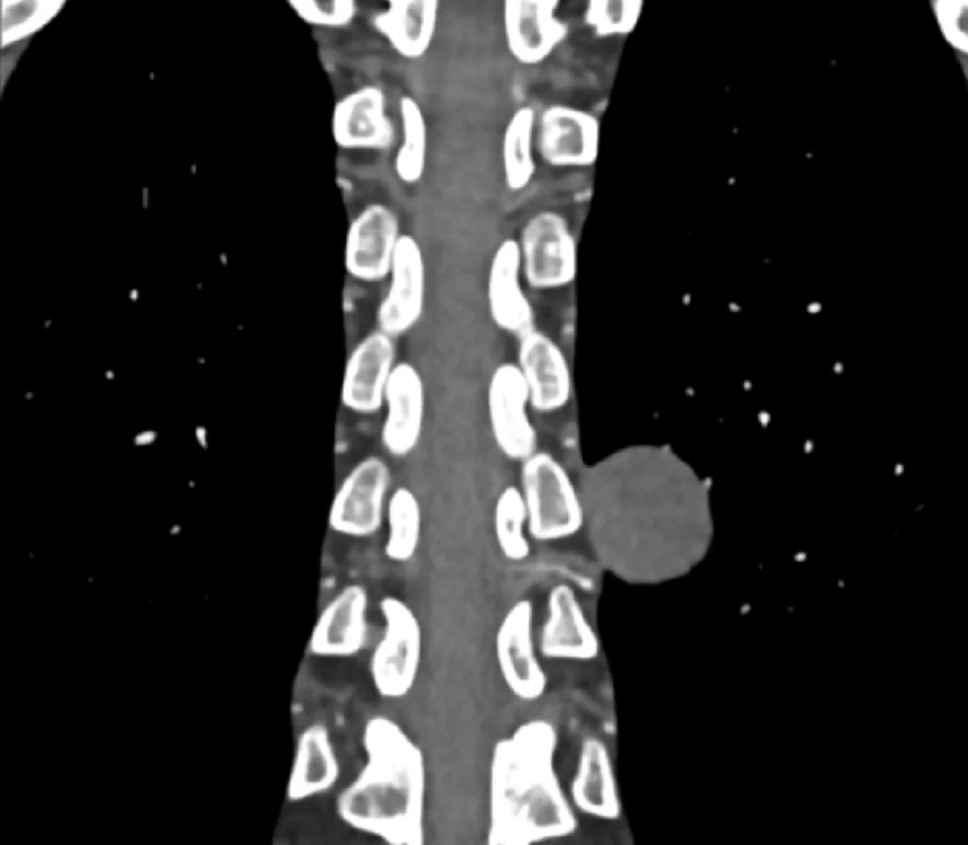
Visualization of the Adamkiewicz artery (arteria radicularis anterior magna) on computed tomography (CT) angiography

As demonstrated by Eguchi et al. in the surgical management of mediastinal masses, recent technological advances particularly artificial intelligence and 3D modeling have begun to play an increasingly important role in enhancing the evaluation of complex anatomical relationships in mediastinal tumors [10] (Fig. 5). However, these methods have not yet surpassed CT angiography in the visualization of the Adamkiewicz artery (arteria radicularis anterior magna), which remains the most reliable modality for this purpose (Fig. 6).

Preoperative fine-needle aspiration biopsy is recommended in patients where a definitive preoperative diagnosis cannot be established or where there is diagnostic uncertainty. This approach helps prevent unexpected intraoperative findings and allows for appropriate surgical planning, including incision type and location. In our series, this technique was rarely required but was implemented in selected cases to enhance patient safety [11].

Schwannomas most commonly originate from the posterior mediastinum; however, atypically located thoracic schwannomas are rare, with only a limited number of case reports in the literature. Wang et al. reported a case of a schwannoma located in the middle mediastinum, while Zhu et al. described one in the superior mediastinum. Oosterwijk et al. presented a case arising from the chest wall, and Abdellatif and Pinilla et al. documented cases of parenchymal schwannomas. Additionally, Righini et al. reported a rare case originating from the tracheal lumen [12–18].

Depending on tumor localization and patient comorbidities, various surgical techniques—such as video-assisted thoracoscopic surgery (VATS), robot-assisted thoracic surgery (RATS), thoracotomy, and modified open approaches can be effectively utilized for complete excision of these tumors [18, 19].

### Surgical considerations in challenging cases

Among the atypical localizations, the most challenging cases involved tumors situated near the brachial plexus, the subclavian vessels, or within the pre-carinal mediastinum. In supraclavicular and infraclavicular lesions, the surgical approach was planned to provide wide exposure of the neurovascular structures. After identifying the main trunks of the brachial plexus, dissection proceeded along the tumor capsule using sharp and blunt techniques, guided by intermittent intraoperative nerve stimulation to prevent traction injury. For masses adjacent to the subclavian artery, proximal and distal vascular control was ensured before mobilization of the lesion. These principles enabled complete resection without neurological or vascular complications.

### Clinical relevance

Atypical thoracic schwannomas may present with non-specific or misleading symptoms and can closely mimic other mediastinal, cervical, or chest wall pathologies. For this reason, clinicians should maintain a high index of suspicion when evaluating well-circumscribed thoracic masses located outside the posterior mediastinum. Careful preoperative imaging particularly MRI for defining neural or vascular relationships plays a decisive role in selecting the safest surgical approach. Our experience demonstrates that even when schwannomas arise in anatomically challenging regions, complete resection can be achieved with minimal morbidity through individualized incision planning and the appropriate use of minimally invasive techniques. Incorporating these considerations into routine practice may facilitate earlier diagnosis, optimize operative strategy, and ultimately improve patient outcomes.

## Conclusion

This case series highlights the importance of considering schwannomas in the differential diagnosis of atypically located thoracic masses. Recognizing this possibility during the preoperative period and conducting appropriate diagnostic evaluations can contribute to accurate surgical planning and favorable patient outcomes. These tumors should be surgically resected in a safe and controlled manner, following thorough evaluation for potential invasion of adjacent structures.

## Data Availability

The datasets generated and/or analyzed during the current study are available from the corresponding author upon reasonable request.
